# Isolation of lytic bacteriophages and their relationships with the adherence genes of *Staphylococcus saprophyticus*

**DOI:** 10.1186/s13104-024-06864-y

**Published:** 2024-07-22

**Authors:** Maryam Rafiee, Alijan Tabarraei, Mahsa yazdi, Ezzat Allah Ghaemi

**Affiliations:** 1 Laboratory Sciences Research Center, Golestan University of Medical Sciences, Gorgan, Iran; 2 Department of Biology, Faculty of Sciences, University of Isfahan, Isfahan, Iran

**Keywords:** *Staphylococcus saprophyticus*, Bacteriophage, Siphoviridae, Wastewater

## Abstract

**Objective:**

This study aimed to introduce a lytic bacteriophage against *Staphylococcus saprophyticus* from wastewater in Gorgan, northern Iran.

**Results:**

The vB_SsapS-46 phage was isolated from urban wastewater and formed round and clear plaques on bacterial culture. It was visualized by electron microscopy and had a large head (approximately 106 nm) and a long tail (approximately 150 nm), indicating that it belongs to the Siphoviridae family. The host range of vB_SsapS-46 was determined using a spot test on 35 *S. saprophyticus* clinical isolates, and it was able to lyse 12 of the 35 clinical isolates (34%). Finally, the relationship between phage sensitivity and adherence genes was assessed, revealing no significant correlation between phage sensitivity and the frequency of adherence genes. The vB_SsapS-46 phage can be used alone or in a mixture in future studies to control urinary tract infections caused by this bacterium, especially in the elimination of drug-resistant pathogens.

## Introduction

*Staphylococcus saprophyticus* has been identified as the second causative agent of uncomplicated urinary tract infections (UTIs) in young females. This bacterium is associated with several complications, including pyelonephritis, cystitis, and in some cases, blood infections. Generally, *S. saprophyticus* is present in humans as part of the normal flora of the skin, perineum, urethra, cervix, rectum, and gastrointestinal tract. In 1961, Torres Pereira isolated a coagulase-negative Staphylococcus carrying 51 antigens from the urine of women with acute UTI. The organism was later classified as part of subgroup 3 of Micrococcus, which was subsequently identified as *S. saprophyticus* [1, 2].

Overall, *S. saprophyticus* isolates are typically considered sensitive to antibiotics commonly used to treat UTIs, as proposed by the Clinical and Laboratory Standards Institute (CLSI) guidelines [3]. The first-line antibiotics typically used are trimethoprim-sulfamethoxazole and nitrofurantoin; however, recent studies have reported resistance to trimethoprim-sulfamethoxazole. This alarming trend requires more attention, highlighting the importance of assessing resistance patterns when choosing antibiotics and necessitating the development of alternative therapies. Thus, it is crucial to select and study the biological properties of new phages against uropathogenic bacteria, particularly *S. saprophyticus*, and create a cocktail phage for the treatment of UTIs [4, 5].

The therapeutic use of phages has several advantages. Recently, bacteriophages have been used as novel antibacterial agents for reducing pathogenic bacteria and their biofilms; therefore, lytic bacteriophages could have various applications as biocontrol agents and in phage therapy [6, 7]. The first study to introduce phages of coagulase-negative staphylococci, including *S. saprophyticus*, by mitomycin C induction was in 1991 [8]. In 2018, in Iran’s Golestan province, the first report of a lytic phage against *S. saprophyticus* (vB_SsapS-104) from hospital wastewater was published [9]. Consequently, given the limited research on bacteriophages of *S. saprophyticus*, a new project was conducted in 2021 using two approaches: isolation and identification of more lytic bacteriophages from urban and hospital wastewater against *S. saprophyticus* due to the bacteria entering the wastewater through urine, and genomic examination of bacteriophages for registration with the International Committee on Taxonomy of Viruses (ICTV). Phages exhibiting lytic activity against multidrug-resistant isolates have attracted considerable attention as alternative antibiotics. The antibacterial activities of phages against staphylococcal species such as *Staphylococcus epidermidis* and *Staphylococcus aureus* have been extensively demonstrated [10–13]. However, only two studies have reported lytic phages against *S. saprophyticus*. The present study aimed to isolate a lytic phage from wastewater against *S. saprophyticus* in Golestan Province, northern Iran.

## Materials and methods

### Bacterial isolation and characterization

Thirty-five *S. saprophyticus* isolates from UTI patients were collected from Landa Laboratories and Moosavi Hospital in Golestan Province, Iran, and were used as hosts for phage isolation from wastewater samples. The *S. saprophyticus* isolates were initially revived and re-identified through biochemical and molecular tests [14].

### Phage isolation

Raw municipal and hospital sewage from Hakim, Sayad Shirazi, Talghani, and Azar hospitals were collected to isolate specific phages in Golestan Province, Iran. The sewage samples were centrifuged at 10,000 × g for 10 min at 4 °C. Subsequently, the supernatant was mixed with Brain Heart Infusion (BHI) broth containing the *S. saprophyticus* isolates. After incubation, the suspension was centrifuged, and the supernatant was filtered using a 0.22 μm filter (Gilson, UK). Following this, 10 µL of the filtrate was mixed with 100 µL of bacterial culture in melted BHI with 0.7% agar and then poured onto a plate of BHI agar. The plates were incubated at 37 °C, and plaques appeared after overnight incubation [15].

### Purification and titration

To obtain a pure suspension, a single plaque was picked with a pipette, transferred to a tube containing a culture of the bacterial host in BHI broth, and then incubated. After 24 h, the mixture was centrifuged and examined for plaque formation using the double-layer agar method. All steps were repeated three times. To determine the titration of phage particles, 100 µL of the phage lysates were serially diluted 10-fold into 900 µL of microtubes containing 900 µL SM buffer (8 mM MgSO4, 50 mM Tris-HCl, 99 mM NaCl, 0.01 mM gelatin, pH 7). Then, 100 µL of the bacterial host culture from each dilution was added to 10 mL of BHI soft agar at 45 °C and poured onto BHI agar. Finally, after 24 h of incubation, the dilution that included 30 to 300 plaques was counted [16].

### Host range determination

The lytic activity and host range were determined using the spot test method. For this purpose, 35 clinical strains of *S. saprophyticus* were cultured on separate plates, and 10 µL of the purified suspension was spotted on the center of each plate and incubated at 37 °C. After incubation, the clear zones that appeared were indicative of susceptibility to phages. These assays were performed on several standard gram-positive and gram-negative strains such as *Pseudomonas aeruginosa 27,853*, *Staphylococcus epidermidis 1435*, *Proteus mirabilis 43,071*, *Escherichia coli 25,922*, *Shigella flexneri 12,022*, *Enterococcus faecalis 29,212*, and *Staphylococcus aureus 25,923* [17].

### Electron microscopy

To study the morphology of phages using Transmission Electron Microscopy (TEM), 10 µL of the concentrated phage suspension was placed onto a copper grid covered with carbon and allowed to absorb for 5 min. The copper grid was then stained with 2% uranyl acetate for 1 min. The copper grid was washed thoroughly with distilled water and air-dried. Finally, the particles were examined using a Philips transmission electron microscope (Philips, Em208s) at the Rastak Lab (Tehran) at 100 kV [17].

### Correlation between virulence factors and sensitivity to the phage

The association between the presence of adhesion genes in bacterial isolates and phage sensitivity was investigated by polymerase chain reaction (PCR) method for the fibronectin-binding autolysin (Aas), surface-associated lipase (Ssp), collagen-binding serine-aspartate-repeat protein (sdrI), and uro-adherence factor A (UafA) genes, as described previously [18]. Some positive samples were sequenced (Macrogen Co., South Korea). In addition, sequences were identified using BLAST and deposited in GenBank (https://www.ncbi.nlm.nih.gov/genbank/).

### Statistical analysis

Data in this study were analyzed using GraphPad Prism 8.3.1 software. A chi-square test was conducted to evaluate the relationship between sensitivity to phage and the presence of surface adhesion proteins. The significance threshold was set at *p* < 0.05.

## Result

### Bacterial identification

All *S. saprophyticus* isolates were recovered from women with UTI aged 4 to 65 years. As expected, the majority of cases (91.3%) were outpatients, and only three *S. saprophyticus* isolates were collected from hospitalized patients. PCR was performed using specific primers to identify the 16SrRNA gene. The sequences were deposited in GenBank. Accession numbers: MW453017,MW453018,MW453019,MW453020,MW453016,MW453021,MW453014, MW453022, MW453023, MW453015,MW453024.

### Isolation of bacteriophage

**Fig. 1:**
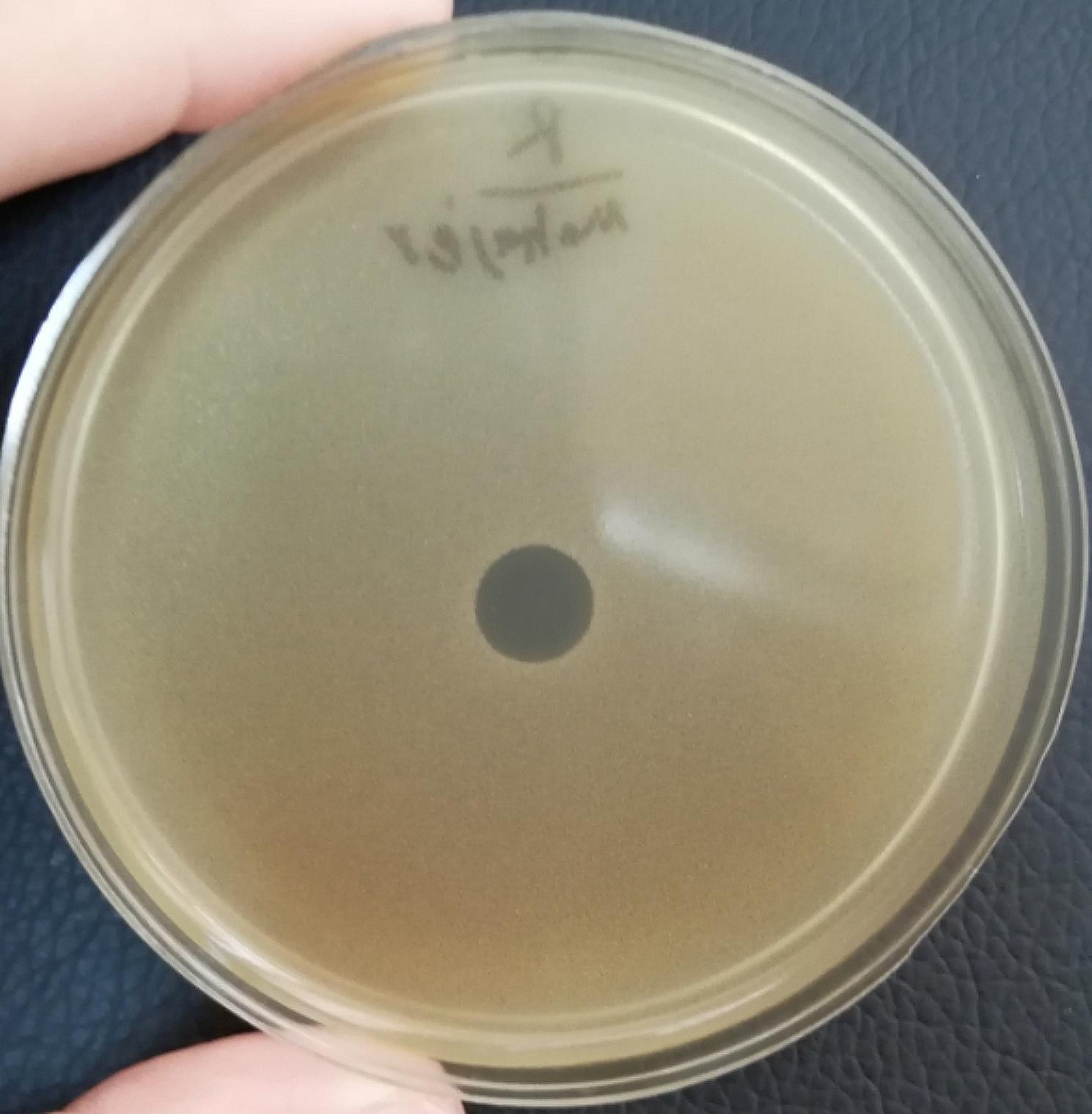
Presence of bacteriophage in the studied samples. Plaque morphology of the vB_SsapS-46 Phage cultured on the *S. saprophyticus* isolate, forming 7 mm plaques without any halo

**Fig. 2:**
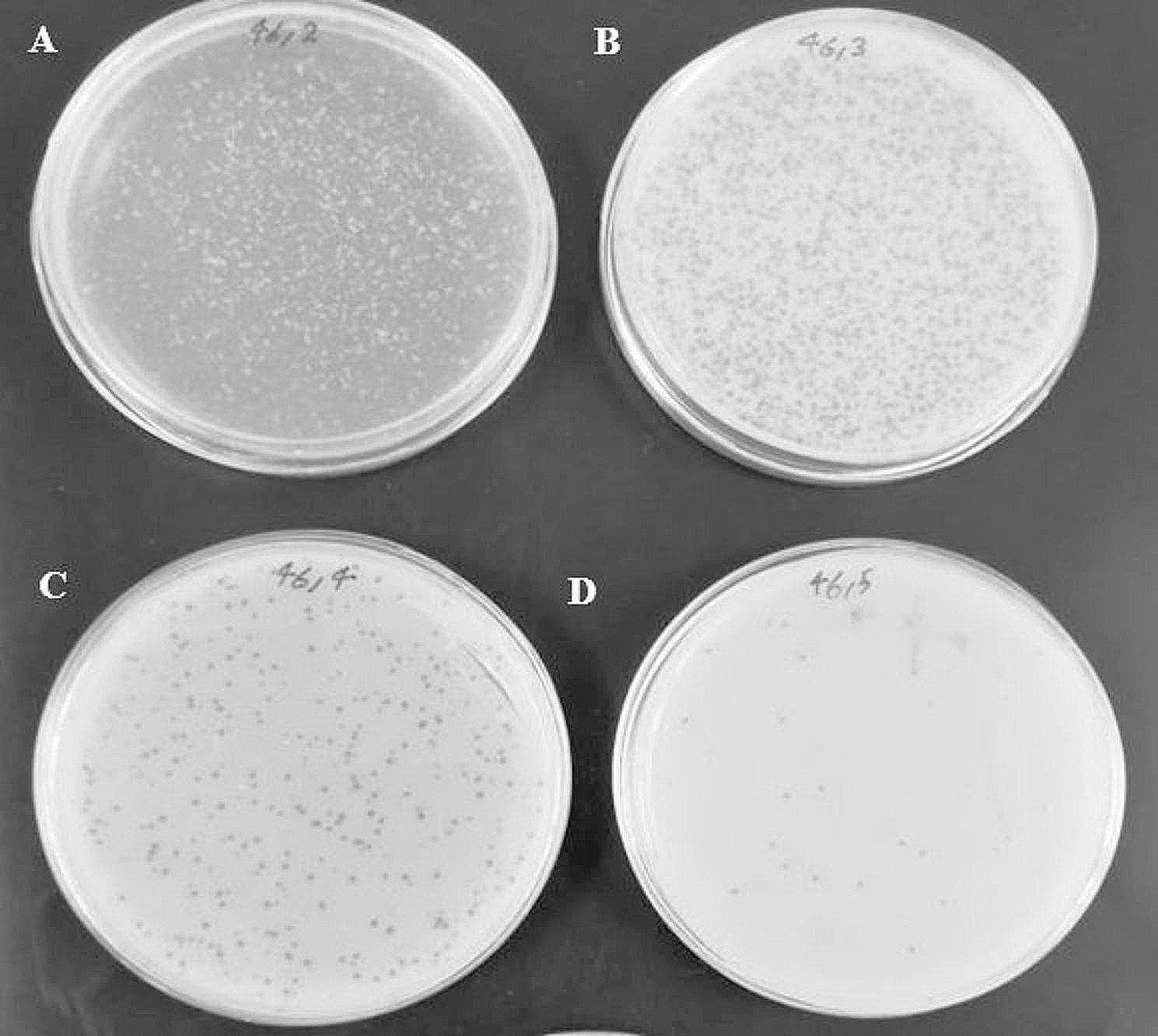
Titration of vB_SsapS-46 phage (**A**) 10^− 2^ dilution, (**B**) 10^− 3^ dilution, (**C**) 10 − ^4^ dilution, and (**D**) 10^-5^ dilution

Lytic bacteriophages were isolated from municipal wastewater using the double-layer technique, and the vB_SsapS-46 phage created plaques with a diameter of 7 mm without a halo in the lawn of *S. saprophyticus* 46; therefore, it was considered the host bacterium. According to Kropinski et al., the phage was named vB_SsapS-46 (Figs. 1 and 2).

### The host range of VB_SsapS-46

The host range of vB_SsapS-46 was determined using 35 *S. saprophyticus* isolates and other clinical strains. The results showed that phage vB_SsapS-46 lysed 12 *S. saprophyticus* isolates (34%), reflecting the host specificity of *S. saprophyticus* isolates. Moreover, vB_SsapS-46 did not infect any other isolates (Table 1).

**Table 1:** Lytic activity of phage VB_SsapS-46 on different bacterial species

Bacterial strain	Plaque formation
*P.aeruginosa27853*	**-**
*S.epidermidis1435*	**-**
*P.mirabilis43071*	**-**
*E.coli25922*	**-**
*S.flexneri12022*	**-**
*E. faecalis29212*	**-**
*S.aureus25923*	**-**

### Morphology of phage VB_SsapS-46

**Fig. 3:**
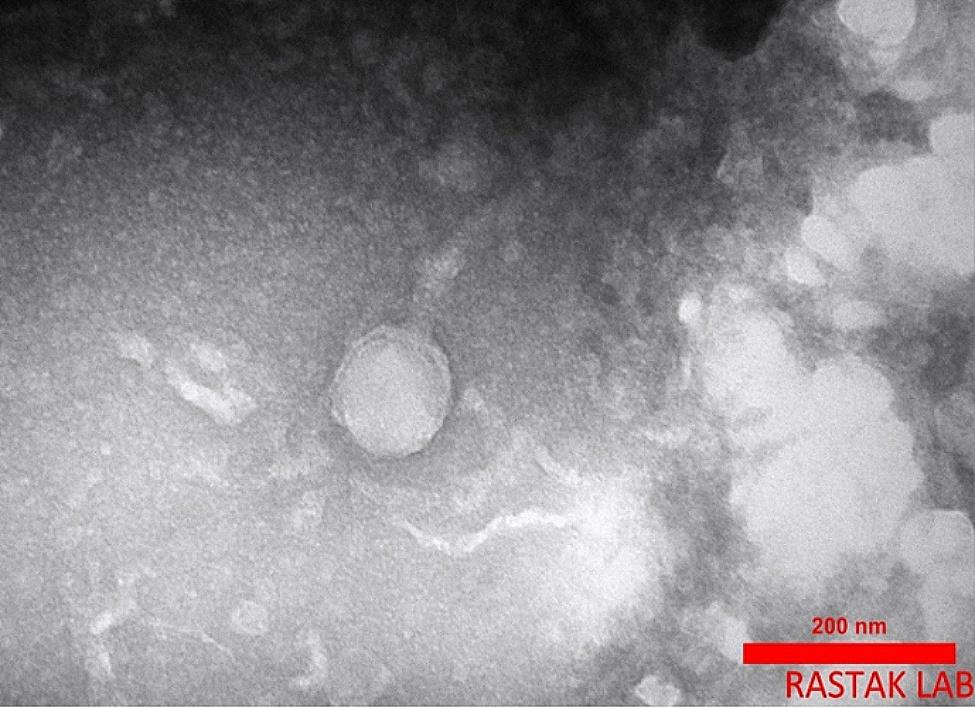
Micrograph of phage VB_SsapS-46 by transmission electron micrograph (TEM). Bar, 200 nm

Transmission electron microscopy analysis indicated that phage particles had an icosahedral head and a tail length with 106 nm and 150 nm diameters, respectively. Based on morphology, the vB_SsapS-46 phage belongs to the Siphoviridae family according to the ICTV classification system (Fig. 3).

### Relation between sensitivity to phage and presence of adherence genes

Table 2 showed that seven adherence gene/spot profiles (A-G) were found. Profile “B” was the most frequent pattern, accounting for 42% (*n* = 15) of all bacterial isolates. Group “G” accounted for 2.8% (*n* = 1) of bacterial isolates. Likewise, there was no significant relationship between sensitivity to phage and the presence of adherence genes (*p* > 0.05) (Table 2). Accession numbers: OP696966, OP696967, OP973206.

**Table 2:** Concurrency of Presence virulence factor genes and phage sensitivity among *S.saprophyticus* isolates

Group	Virulence genes /Spot	No. (%) of isolates
A	*Aas* ^*+*^_,_ *Ssp* ^*+*^_,_ *UafA* ^*+*^ */+*	10 (28.5%)
B	*Aas* ^*+*^_,_ *Ssp* ^*+*^_,_ *UafA* ^*+*^ */-*	15 (42%)
C	*Aas* ^*+*^_,_ *UafA* ^*+*^ */+*	2 (5.7%)
D	*Aas* ^*+*^_,_ *UafA* ^*+*^ */-*	2 (5.7%)
E	*Aas*^*+*^_,_*Ssp*^*+*^/*-*	3 (8.5%)
F	*UafA* ^*+*^ */-*	2 (5.7%)
G	*-/-*	1 (2.8%)

## Discussion

UTI are among the most common human infections. It is estimated that 150 million people are affected annually by UTI. Further, 17–29 $ are spent on the treatment and recovery of hospital infections, of which 39% are related to the costs caused by UTIs every year [19, 20].

*S. saprophyticus* is a coagulase-negative staphylococcus that causes uncomplicated UTI in young and sexually active women. In addition, more than half of women are colonized by this bacterium in the rectum and urethra [1]. *S. saprophyticus* expresses surface proteins that attach to the uroepithelium and secretes various enzymes that can survive in the urinary environment [21].

In this study, the specific and single-species lytic phage VB_SsapS-46 against *S. saprophyticus* was isolated from municipal wastewater. In a study by Elizbarashvili et al., phages vB_S.s. 1 and vB_S.s. 2 against *S. saprophyticus* were isolated from rivers in Georgia [22]. According to Yazdi et al., phage vB_SsapS-104 is effective against this bacterium obtained from hospital wastewater in Iran [9]. These findings show that the isolated bacteriophages are highly stable and active in various aqueous environments such as hospitals, municipal sewage, and rivers. Hence, they can easily survive under various conditions, such as different temperatures, chemicals, and rays.

Determining the host range helps define the biological characteristics of the phage and is a critical step in phage therapy. The host range of our isolated phage was examined on 35 clinical *S. saprophyticus* strains by the spot test. Phage VB_SsapS-46 was able to attack 12 clinical strains (34%) of *S. saprophyticus*. Elizbarashvili et al. and Yazdi et al. found that *S. saprophyticus* bacteriophages could also lyse strains (9%) and (88.8%), respectively [9, 22]. More than 95% of isolated phages belong to the order Caudovirales. Most phages with non-contractile tails are related to the family Siphoviridae. Electron microscopy surveying showed that the isolated phage belongs to the Siphoviridae family, consistent with previous studies results [9, 22].

Numerous factors, such as the infectious capacity of the organism, host interaction, and amount of inoculum, interact to cause infection. Colonization is the first event that leads to the onset of infection, and uropathogenic bacteria such as *S. saprophyticus* have proteins attached to their cell surface [1, 23]. These adhesions, essential for the initial attachment, mediate binding to tissue receptors and uroepithelial cells. The first stage of bacteriophage infection involves the adsorption of receptors on the bacterial cell surface. Phages can bind to these surface receptors and enter and lyse bacterial hosts [24]. Previous study assessed at least four virulence factor genes related to urinary tract colonization by *S. saprophyticus* [18, 21].

The most common virulence gene detected in these isolates was Aas, which is a multifunctional protein with adhesive properties that binds to fibronectin. The next most frequent virulence gene was UafA, which mediates adherence to human bladder epithelial cells [18]. Findings suggest that there is no direct correlation between these surface receptors and the capacity to enter the bacterial host. The VB_SsapS-46 phage may utilize other cell surface proteins (which also serve as phage receptors) within the bacterial host.We found one *S. saprophyticus* isolate (Table 2, Group G), which does not have any adherence gene and is resistant to the VB_SsapS-46 phage; therefore, complete sequencing of this isolate is interesting and should be performed in future studies.

## Conclusion

While various studies of phages infecting Staphylococcus bacteria have received considerable attention, those infecting *S. saprophyticus* have been less thoroughly investigated. This study introduced phage vB_SsapS-46 as a biocontrol to prevent the growth of *S. saprophyticus*, which causes UTI. Based on morphology, the isolated phage belongs to the Siphoviridae family. Its specificity for *S. saprophyticus* isolates emphasizes the potential of phage vB_SsapS-46 for therapeutic use. However, further research on the antibacterial application of this phage in vivo is required.

### Limitations

A limitation of this study may be the difficult and time-consuming isolation of the phage.

## Data Availability

The accession numbers of the deposited genes in GenBank were added in the Availability of Data section.
